# A Divergent Alkyne Diol Directs [2 + 2] Photoreactivity in the Solid State: Cocrystal, Supramolecular Catalysis, and Sublimation Effects

**DOI:** 10.3390/molecules24173059

**Published:** 2019-08-22

**Authors:** Shalisa M. Oburn, Jay Quentin, Leonard R. MacGillivray

**Affiliations:** Department of Chemistry, University of Iowa, Iowa City, IA 52242, USA

**Keywords:** supramolecular catalysis, divergent template, solid state, [2 + 2] photocycloaddition

## Abstract

2-butyne-1,4-diol (**1,4-bd**) is used as a divergent ditopic template that directs *trans*-1,2-bis (*n-*pyridyl) ethylene (***n,n*′-bpe**, where *n* = *n*′ = 3 or 4) to undergo an intermolecular [2 + 2] photodimerization in the solid state. The components of cocrystals [(**1,4-bd**)·(**4,4′-bpe**)]_n_ and [(**1,4-bd**)·(**3,3′-bpe**)]_n_ form 1D hydrogen-bonded polymers with ***n*,*n*′-bpe** assembled as infinite parallel stacks. The alkenes undergo [2 + 2] photocycloadditions to form *rctt*-tetrakis (*n*-pyridyl) cyclobutane (where *n* = 3 or 4). We demonstrate that the reactive solid involving **4,4′-bpe** exhibits supramolecular catalysis.

## 1. Introduction

We have recently demonstrated that intermolecular [2 + 2] photodimerizations can be directed using principles of supramolecular catalysis in the solvent-free environment of the solid state. The ditopic hydrogen-bond-donor 4,6-dichloro-resorcinol (**4,6-diCl-res**) was used in catalytic amounts as a template to achieve a [2 + 2] photodimerization of *trans*-1,2-bis (4-pyridyl) ethylene (**4,4′-bpe**) [1,2]. Supramolecular catalysis of the photodimerization involving **4,4′-bpe** was also realized using ditopic organoboronic acids as templates [3]. The approach to supramolecular catalysis was performed mechanochemically via vortex grinding [2]. In both cases, the catalysts functioned as convergent ditopic receptors with reactivity occurring within zero-dimensional (0D), or discrete, hydrogen-bonded structures.

Seminal work of Toda has reported on the reactivity properties of host–guest complexes formed with the divergent ditopic host 1,1,6,6-tetraphenyl-hexa-2,4-diyne-1,6-diol (**1,6-diol**). The diol interacted with chalcones to generate photoactive hydrogen-bonded solids [4]. The **1,6-diol** assembled the olefins within solid-state cavities supported by the bulky tetraphenyl-substituents of the host. The reactive solids based on **1,6-diol** also formed via dry grinding [5]. Moreover, simple shaking of 1:2 and 1:4 solid mixtures of **1,6-diol** and the chalcones resulted in up to 90% photoconversion following UV-irradiation.

**Scheme 1:**
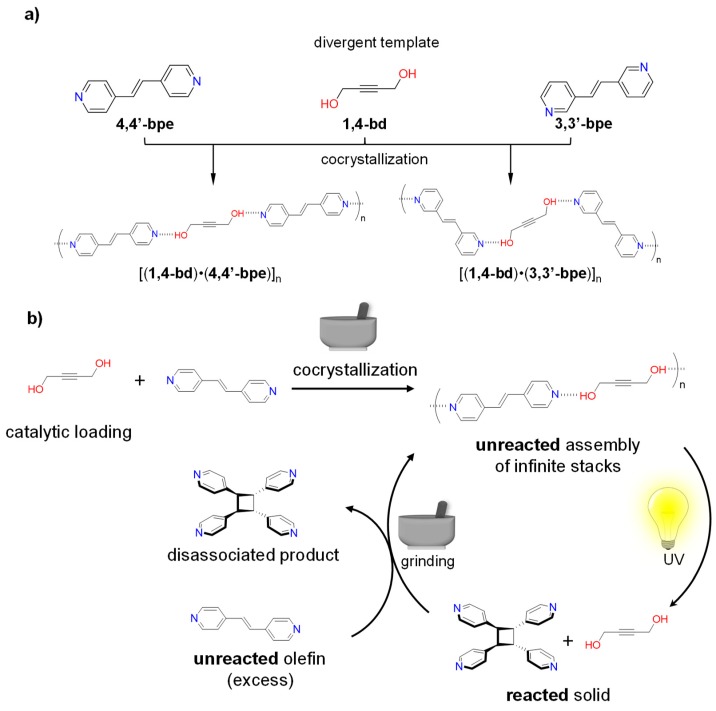
Photoreactive cocrystals using (**a**) divergent **1,4-bd** and (**b**) supramolecular catalysis.

Here, we report the ability of 2-butyne-1,4-diol (**1,4-bd**)—effectively, a minimalist derivative of **1,6-diol**—to act as a divergent ditopic template of an intermolecular [2 + 2] photodimerization in the solid state (Scheme 1). Cocrystallizations of **1,4-bd** with *trans*-1,2-bis (*n*-pyridyl) ethylene (***n*,*n*′-bpe**, where *n* = *n*′ = 3 or 4) generate cocrystals of composition [(**1,4-bd**)·(**4,4′-bpe**)]_n_ and [(**1,4-bd**)·(**3,3′-bpe**)]_n_. The components of the cocrystals form 1D hydrogen-bonded polymers with olefins that stack into infinite columns. UV-irradiation of each solid generates *rctt*-tetrakis (*n*-pyridyl) cyclobutanes (***n,n*′-tpcb**) stereoselectively. We also reveal **1,4-bd** to act as a supramolecular catalyst for the photoreaction involving **4,4′-bpe**.

## 2. Results and Discussion

### 2.1. Photoreactive Cocrystals of *[(**1,4-bd**)·(**4,4′-bpe**)]_n_*

Cocrystals of composition [(**1,4-bd**)·(**4,4′-bpe**)]_n_ were obtained by combining equimolar solutions of **4,4′-bpe** and **1,4-bd** in acetonitrile, and allowing the resulting solution to slowly evaporate to yield colorless plate-like crystals suitable for single-crystal X-ray diffraction.

**Figure 1:**
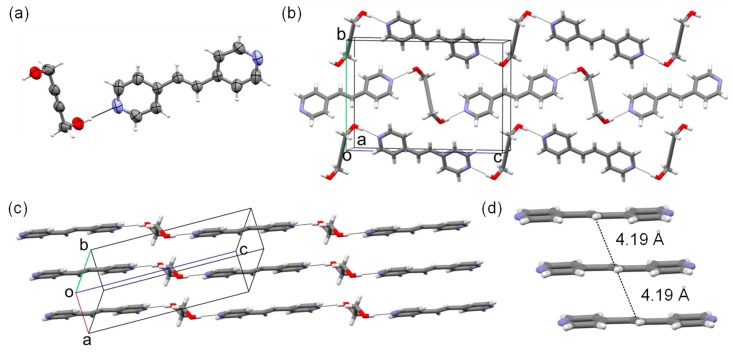
X-ray structure [(**1,4-bd**)·(**4,4′-bpe**)]_n_: (**a**) ORTEP representation, (**b**) 1D hydrogen-bonded chains, (**c**) stacks of hydrogen-bonded chains, (**d**) stacked C = C bonds of **4,4′-bpe**.

The components of [(**1,4-bd**)·(**4,4′-bpe**)]_n_ crystallize in the monoclinic space group *P*2_1_/*c* (Table 1, Figure 1). The asymmetric unit contains one half each of a molecule of **1,4-bd** and **4,4′-bpe**. The diol and bipyridine interact via O-H···N hydrogen bonds [O(1)···N(2) separation (Å): 2.792(2)] to generate infinite 1D hydrogen-bonded chains that propagate along the crystallographic *c*-axis. The hydroxyl groups of **1,4-bd** adopt an *anti*-conformation with a dihedral angle of nearly 180°. Adjacent 1D hydrogen-bonded chains are sustained by bifurcating-type C-H(pyridine)···O and C-H(alkene)···O forces along the *b*-axis [C···O separations (Å): C(8)···O(1) 3.43, C(4)···O(1) 3.58] such that neighboring 1D chains lie offset and tilted by 56.4°. The hydrogen-bonded chains pack into segregated 2D stacks along the *a*-axis. The carbon–carbon double (C = C) bonds of the stacked olefins are organized parallel and separated by 4.19 Å, which conforms to the criteria of Schmidt for a [2 + 2] photocycloaddition in the solid state [6].

To determine photoreactivity of [(**1,4-bd**)·(**4,4′-bpe**)]_n_, a finely-ground crystalline powder was distributed between two glass plates and exposed to UV-irradiation (450 W medium-pressure Hg lamp) for a period of 55 h. A ^1^H-NMR spectrum revealed the emergence of a single cyclobutane resonance (4.68 ppm) consistent with *rctt*-tetrakis (4-pyridyl) cyclobutane (**4,4-tpcb**) (79% conversion), which was accompanied by a decrease in intensity of the olefinic signals (see Figure S2). Prolonged UV exposure did not result in an increase in conversion of olefin to cyclobutane. We also note that the amount of **1,4-bd** present in the sample decreased by 6% during the photoreaction. We ascribe the decrease in **1,4-bd** to sublimation of the diol, which may also affect the overall photoconversion. Indeed, we have determined that **1,4-bd** as a pure form sublimes at 40–45 °C at ambient pressure. Sublimation of **1,4-bd** from the unreacted cocrystal, however, does not occur (see Experimental). We note that Groeneman has recently reported that the template 1,2-dibromo-4,5-diflorobenzene can be sublimed for product purification [7]. We confirmed the stereochemistry of the photoproduct generated from [(**1,4-bd**)·(**4,4′-bpe**)]_n_ by treatment of acid and extraction into chloroform (see Figure S3) [8].

### 2.2. Photoreactive Cocrystals of *[(**1,4-bd**)·(**3,3′-bpe**)]_n_*

Cocrystals of [(**1,4-bd**)·(**3,3′-bpe**)]_n_ were obtained by dissolving equimolar **3,3′-bpe** and **1,4-bd** in minimal boiling diethyl ether. The solvent was allowed to slowly evaporate for 2 d to yield colorless single crystals of [(**1,4-bd**)·(**3,3′-bpe**)]_n_. In contrast to [(**1,4-bd**)·(**4,4′-bpe**)]_n_, the cocrystals [(**1,4-bd**)·(**3,3′-bpe**)]_n_ were determined to be highly deliquescent.

**Figure 2:**
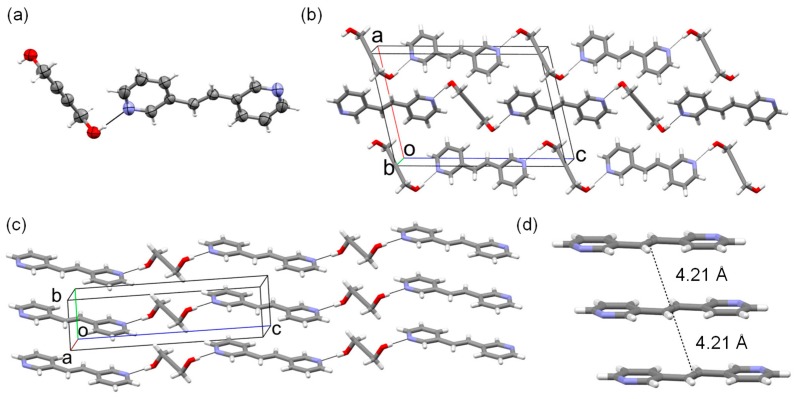
X-ray structure [(**1,4-bd**)·(**3,3′-bpe**)]_n_: (**a**) ORTEP representation; (**b**) 1D chains; (**c**) stacks of hydrogen-bonded chains; (**d**) stacked C = C bonds of **3,3′-bpe**.

The components of [(**1,4-bd**)·(**3,3′-bpe**)]_n_ crystallize in the monoclinic space group *P*2_1_/*n* (Table 1, Figure 2). Similar to [(**1,4-bd**)·(**4,4′-bpe**)]_n_, the asymmetric unit of [(**1,4-bd**)·(**3,3′-bpe**)]_n_ consists of one half of a molecule of **1,4-bd** and **3,3′-bpe**. The components assemble via O-H···N hydrogen bonds [O(1)···N(2) separation (Å): 2.802(1)] to form infinite 1D hydrogen-bonded chains along the crystallographic *c*-axis. The hydroxyl groups of **1,4-bd** also adopt an *anti*-conformation with a dihedral angle of nearly 180°. Adjacent 1D chains are sustained by bifurcating C-H(pyridine)···O and C-H(alkene)···O forces along the *a*-axis [C···O separations (Å): C(8)···O(1) 3.45, C(5)···O(1) 3.56] such that neighboring chains lie offset and tilted by 61.6°. The hydrogen-bonded chains arrange into infinite offset 2D stacks along the *b*-axis such that the C = C bonds lie parallel and separated by 4.21 Å.

When a finely-ground crystalline powder of [(**1,4-bd**)·(**3,3′-bpe**)]_n_ in a 20-mL scintillation vial was exposed to UV-radiation for 23 h, a ^1^H-NMR spectrum revealed **3,3′-bpe** to be converted a cyclobutane product (68% conversion) (see Figure S5). The formation of the photoproduct was evidenced by the emergence of a single cyclobutane resonance (4.71 ppm) consistent with *rctt*-tetrakis (3-pyridyl) cyclobutane (**3,3′-tpcb**) and a concomitant decrease in intensity of the olefinic resonances

The stereochemistry of the photoproduct **3,3′-tpcb** as *rctt-* was confirmed by treatment with acid and extraction into chloroform (see Figure S6). Single crystals of (*rctt*-**3,3′-tpcb**)·(H_2_O) in the form of plates were obtained by slow solvent evaporation over a period of 4 d in chloroform.

**Figure 3:**
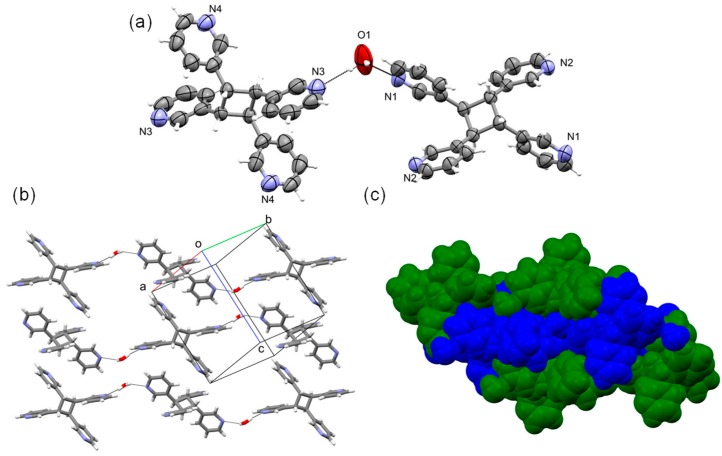
X-ray structure (*rctt*-**3,3′-tpcb**)·(H_2_O): (**a**) ORTEP representation; (**b**) 1D hydrogen-bonded chains (**c**) interdigitated 2D sheets with separate 2D sheets blue and green.

The components of (*rctt*-**3,3′-tpcb**)·(H_2_O) crystallize in the triclinic space group $P\bar{1}$ (Table 1). The asymmetric unit consists of two unique one-half molecules of *rctt*-**3,3′-tpcb** and one full molecule of H_2_O. One cyclobutane (N3/N4) lies disordered over two positions (occupancies C17A/C17B 70/30). The *transoid*-pyridyl groups of the cyclobutanes adopt *anti*- (N1/N2) and *syn*-conformations (N3/N4) (Figure 3). The components of the solid assemble via O-H···N hydrogen bonds [O···N separations (Å): 2.926(6) O(1)/N(1), 2.839(5) O(1)/N(3)] with the included water molecules bridging adjacent cyclobutanes. As a consequence of the assembly process, the components form 1D hydrogen-bonded chains along the crystallographic *b*-axis. Adjacent 1D chains stack along the *a*-axis and interact via edge-to-face C-H···*π* contacts to form 2D sheets. The 2D sheets interdigitate perpendicular to the *c*-axis.

### 2.3. Mechanochemistry and Supramolecular Catalysis

**Figure 4:**
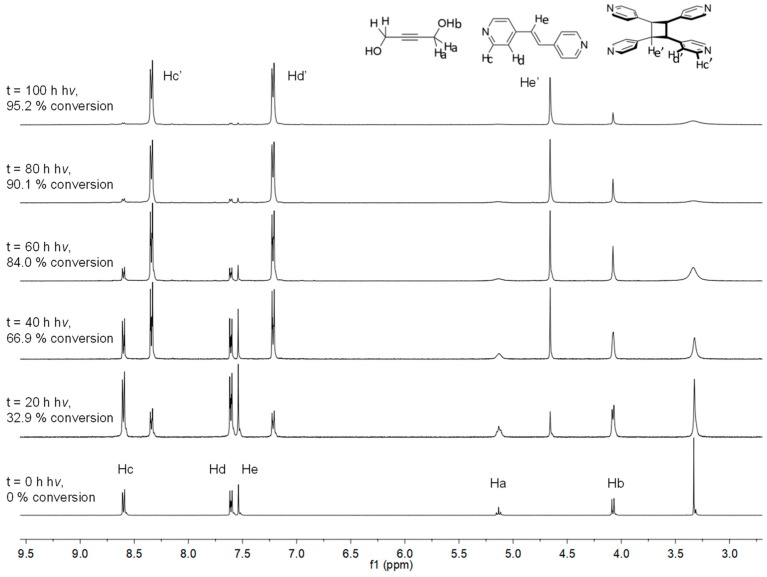
^1^H-NMR spectra (300 MHz, DMSO-*d*_6_) monitoring photoreactivity of [(**1,4-bd**)·(**4,4′-bpe**)]_n_ at 50% catalyst loading of **1,4-bd** (100 h UV exposure). UV-exposure time (t) indicated with stacked spectra.

The diol **1,4-bd** acts as a supramolecular catalyst to form **4,4′-tpcb** in near quantitative yield. Specifically, dry-grinding of equimolar **1,4-bd** and **4,4′-bpe** using an agate mortar and pestle generated the cocrystal [(**1,4-bd**)·(**4,4′-bpe**)]_n_ in 20 min (see Figure S8). Moreover, cycles (5 cycles) of mechanical grinding followed by exposure to UV-radiation (20 h) with 50% loading of **1,4-bd** resulted in near quantitative (95%) conversion of **4,4′-bpe** to **4,4′-tpcb** (Figure 4, see Figure S9). Similar results were obtained for 20% loading of **1,4-bd**, albeit with lower (34%) conversion. ^1^H-NMR assay suggested **1,4-bd** to sublime under the mechanochemical conditions in both cases (see Figure S10) [7]. PXRD data was consistent with crystalline [(**1,4-bd**)·(**4,4′-bpe**)]_n_ and **4,4′-tpcb** being generated during the catalysis and grinding (see Figure S11) [1].

Infinitely stacked C = C bonds in the solid state will exhibit a maximum possible theoretical conversion owing to independent photodimerization events taking place [9,10,11,12] We envision that the increase in conversion of **4,4′-bpe** to **4,4′-tpcb** achieved by supramolecular catalysis relative to the stoichiometric cocrystal [(**1,4-bd**)·(**4,4′-bpe**)]_n_ likely involves ‘free’ diol being available for recrystallization to support reactivity of the C = C bonds between the 1D hydrogen-bonded chains.

## 3. Conclusions

We have demonstrated **1,4-bd** to function as a divergent template to direct [2 + 2] photodimerizations in the solid state. Cocrystals containing **1,4-bd** generate 1D hydrogen-bonded chains of [(**1,4-bd**)·(***n*,*n*′-bpe**)]_n_ (where: *n* = *n*′ = 3 or 4) that form infinite stacks. The cocrystals undergo [2 + 2] photodimerizations to form *rctt*-***n,n*′-tpcb** (*n* = *n*′ = 3 or 4), with sublimation effects involving the template. An increase in yield of the photodimerization of [(**1,4-bd**)·(**4,4′-bpe**)]_n_ is realized with supramolecular catalysis. Our current efforts are focused to identify and apply approaches involving divergent templates to alkenes of increasing structural complexity.

## 4. Materials and Methods

### 4.1. Materials

Reagents 1,4-butynediol (**1,4-bd**) and *trans*-1,2-bis (4-pyridyl) ethylene (**4,4′-bpe**) were purchased from Sigma Aldrich^©^ (St. Louis, MO, USA). Anhydrous diethyl ether (Et_2_O) and acetonitrile (MeCN) were purchased from Fisher Scientific^TM^ and VWR International^©^, respectively. Chloroform (CHCl_3_) and dichloromethane (CH_2_Cl_2_) were purchased from Fischer Scientific^TM^. All chemicals were used as received without further purification. The alkene *trans*-1,2-bis (3-pyridyl) ethylene (**3,3′-bpe**) was synthesized via a Hiyama–Heck coupling of triethoxy(vinyl)silane and 3-bromopyridine [13].

### 4.2. Cocrystallizations

Cocrystals of [(**1,4-bd**)·(**4,4′-bpe**)]_n_ were obtained by dissolving equimolar **4,4′-bpe** (346 mg, 2.0 mmol) and **1,4-bd** (172 mg, 2.0 mmol) separately in MeCN (10 mL). The two solutions were combined and the solvent was allowed to slowly evaporate for 6 d to yield colorless plate-like crystals of [(**1,4-bd**)·(**4,4′-bpe**)]_n_ suitable for single-crystal X-ray diffraction (SCXRD). ^1^H-NMR (300 MHz, DMSO-*d*_6_): δ 8.60 (dd, *J* = 4.5, 1.6 Hz, 2H), 7.61 (dd, *J* = 4.5, 1.6 Hz, 2H), 7.55 (s, 1H), 5.29 − 5.02 (m, 1H), 4.22 − 3.95 (m, 2H).

For synthesis of [(**1,4-bd**)·(**3,3′-bpe**)]_n_, equimolar **3,3′-bpe** (39 mg, 0.2 mmol) and **1,4-bd** (19 mg, 0.2 mmol) were dissolved together in minimum boiling Et_2_O. The solvent was allowed to slowly evaporate for 2 d to yield colorless plate-like crystals of [(**1,4-bd**)·(**3,3′-bpe**)]_n_ suitable for SCXRD. ^1^H-NMR (300 MHz, DMSO-*d*_6_): δ 8.79 (d, *J* = 2.2 Hz, 1H), 8.49 (d, *J* = 4.7 Hz, 1H), 8.07 (d, *J* = 8.0 Hz, 1H), 7.43 (s, 1H), 7.43 (t, *J* = 4.8 Hz, 1H), 5.20 − 5.04 (m, 1H), 4.08 (dd, *J* = 5.9, 0.7 Hz, 2H). Cocrystals of [(**1,4-bd**)·(**3,3′-bpe**)]_n_ were determined to be highly deliquescent.

### 4.3. Photodimerization

All single crystals were finely ground using a mortar and pestle and then spread thinly between a pair of Pyrex glass plates for [(**1,4-bd**)(**4,4′-bpe**)]_n_. Finely ground crystals of [(**1,4-bd**)(**3,3′-bpe**)]_n_ were thinly spread across the bottom of a 20-mL glass scintillation vial. The samples were irradiated in 12 h intervals (ACE Glass photochemistry cabinet, 450 W, medium-pressure Hg-vapor lamp). Cyclobutane formation was monitored using ^1^H-NMR spectroscopic assay.

### 4.4. Photoproduct Isolation

A photoreacted solid of [(**1,4-bd**)·(**4,4′-bpe**)]_n_ was dissolved in 3 M HCl and subsequently washed with CH_2_Cl_2_ (3 × 15 mL). The photoproduct was precipitated from the aqueous solution using 50% NaOH to pH 12 and extracted with CHCl_3_ (3 × 15 mL). The organic layer was dried (Na_2_SO_4_) and discoloration was removed using activated carbon. The solution was filtered and solvent was removed under vacuum to yield *rctt*-tetrakis (4-pyridyl) cyclobutane (*rctt*-**4,4-tpcb**) [8]. ^1^H-NMR (300 MHz, DMSO-*d*_6_): δ 8.34 (dd, *J* = 4.5, 1.5 Hz, 2H), 7.22 (dd, *J* = 4.5, 1.6 Hz, 2H), 4.66 (s, 1H).

A photoreacted solid of [(**1,4-bd**)·(**3,3′-bpe**)]_n_ was dissolved in 3 M HCl and subsequently washed with CH_2_Cl_2_ (3 × 15 mL). The photoproduct was precipitated from the aqueous solution using 50% NaOH to pH 11 and extracted with CHCl_3_ (3 × 15 mL). The organic layer was dried (Na_2_SO_4_). The solution was filtered and solvent evaporated under vacuum. The crude product was purified using flash column chromatography. The column was primed with 10% MeOH/CH_2_Cl_2_. The percentage of MeOH in CH_2_Cl_2_ was increased in 2% increments until pure photoproduct was collected. Fractions containing photoproduct were combined and solvent was removed under vacuum to yield *rctt*-tetrakis (3-pyridyl) cyclobutane (*rctt*-**3,3-tpcb**). ^1^H-NMR (300 MHz, CDCl_3_): δ 8.37 (d, *J* = 2.1 Hz, 1H), 8.31 (dd, *J* = 4.8, 1.4 Hz, 1H), 7.42 − 7.30 (m, 1H), 7.06 (dd, *J* = 7.9, 4.8 Hz, 1H), 4.52 (s, 1H).

### 4.5. X-ray Diffraction Experiments

Diffraction data were collected on a Bruker^©^ Nonius^©^ (Billerica, MA, USA) APEX II Kappa single-crystal X-ray diffractometer at room temperature (298.15 K) using graphite-monochromated Mo Kα_1_ radiation (*λ* = 0.71073 Å). Structure solution and refinement were accomplished using ShelXT [14] and ShelXL [15], respectively, in the Olex2 user graphical interface. The structures were solved using direct methods. All non-hydrogen atoms were identified from the difference Fourier map and refined anisotropically. All hydrogen atoms were placed in their calculated positions and were refined using isotropic thermal parameters.

All powder samples were mounted on glass slides. Each sample was finely ground using an agate mortar and pestle prior to mounting. PXRD data were collected by a Siemens D5000 X-ray diffractometer using Cu Kα_1_ radiation (*λ* = 1.54056 Å) (scan type: locked coupled; scan mode: continuous; step size: 0.02°). PXRD data of (*rctt*-**3,3′-tpcb**)·(H_2_O) were collected at room temperature on a Bruker D8 Advance X-ray diffractometer. Instrument parameters: radiation wavelength, Cu Kα_1_ (λ = 1.5418 Å); scan type, coupled 2Theta/Theta; scan mode, continuous PSD fast; scan range, 5–40° two-theta; step size, 0.02°; voltage, 40 kV; current, 30 mA. Background subtractions were applied to all experimentally collected data within the Bruker^©^ DIFFRAC.EVA v3.1 software suite.

### 4.6. H-NMR Experiments

All ^1^H-NMR spectra were obtained on a Bruker^©^ Fourier-300 NMR spectrometer (Billerica, MA, USA) operating at 300 MHz. All data were processed with the MestReNova^TM^ v6.0.2 software program.

### 4.7. Mechanochemistry and Catalysis

Cocrystals of [(**1,4-bd**)·(**4,4′-bpe**)]_n_ were generated by combining **1,4-bd** (21.9 mg, 0.3 mmol) and **4,4′-bpe** (48.8 mg, 0.3 mmol) and grinding in agate mortar and pestle for 12 min. Cocrystals of [(**1,4-bd**)·(**3,3′-bpe**)]_n_ were generated by combining **1,4-bd** (48.7 mg, 0.6 mmol) and **3,3′-bpe** (101.6 mg, 0.6 mmol) together and grinding in an agate mortar and pestle for 30 min. The resulting crystalline phases were confirmed using powder X-ray diffraction (PXRD).

For catalysis employing a **1,4-bd** template, **4,4′-bpe** (116 mg, 0.64 mmol) with 50 or 20 mol.% of **1,4-bd** were combined and finely ground using an agate mortar and pestle. The formation of cocrystalline material was confirmed using PXRD. The finely ground crystalline powders were spread thinly between two glass plates and exposed to broadband UV-irradiation. The crystalline materials were exposed to additional dry grinding (20 min in an agate mortar and pestle) after 20 h intervals of UV-irradiation. Solid-state catalysis reactions were monitored using ^1^H-NMR spectroscopy and PXRD.

### 4.8. Sublimation Experiments

A crystalline sample of **1,4-bd** (see ref. [16] for crystal structure) was placed in a glass sublimation apparatus. The apparatus was gently heated to ca. 40 °C and the temperature was maintained for 12 h. The sublimed material was confirmed to be **1,4-bd** by ^1^H-NMR spectroscopic analysis (see Figure S12). ^1^H-NMR (300 MHz, CDCl_3_): δ 4.34 (s, 4H).

## Figures and Tables

**Table 1:** Crystallographic data for [(**1,4-bd**)·(**4,4′-bpe**)]_n_, [(**1,4-bd**)·(**3,3′-bpe**)]_n_, and (*rctt*-**3,3′-tpcb**)·(H_2_O).

Cocrystal/Hydrate	[(1,4-bd)·(4,4′-bpe)]_n_	[(1,4-bd)·(3,3′-bpe)]_n_	(*rctt*-3,3′-tpcb)·(H_2_O)
Formula	C_16_H_16_N_2_O_2_	C_16_H_16_N_2_O_2_	C_24_H_22_N_4_O
Formula weight (g·mol^−1^)	268.31	268.31	382.45
Crystal system	Monoclinic	Monoclinic	Triclinic
Space group	*P*2_1_/*c*	*P*2_1_/*n*	*P*-1
*a* (Å)	4.1905 (4)	10.8899 (12)	8.7650 (9)
*b* (Å)	10.9558 (11)	4.2077 (4)	9.4600 (9)
*c* (Å)	15.8790 (16)	16.2118 (16)	13.0903 (13)
α (°)	90	90	92.879 (5)
β (°)	91.420 (5)	101.580 (5)	94.862 (5)
γ (°)	90	90	112.923 (5)
Z	2	2	2
*V* (Å^3^)	728.79 (12)	727.73 (13)	991.99 (17)
*ρ*_calcd_ (g·cm^−3^)	1.223	1.224	1.280
*T* (K)	298	298	298
μ (mm^−1^)	0.082	0.082	0.081
Reflections collected	10144	9896	7864
*F*(000)	284	284	404
Crystal size (mm^3^)	0.19 × 0.18 × 0.05	0.32 × 0.27 × 0.13	0.34 × 0.22 × 0.07
Independent reflections	1320	1428	4189
Data/restraints/parameters	1320/0/92	1428/0/100	4189/0/284
*R*_int_	0.0257	0.0234	0.0355
*R*_1_ (*I* ≥ 2*σ*(*I*))	0.0361	0.0356	0.0561
*wR* (*F*^2^) (*I* ≥ 2*σ*(*I*))	0.0880	0.0881	0.1293
*R*_1_ (all data)	0.0538	0.0495	0.1277
*wR* (*F*^2^) all data	0.0996	0.0975	0.1618
Goodness-of-fit on *F*^2^	1.054	1.077	1.018
CCDC deposition number	1,942,417	1,942,416	1,871,324

## Supplementary Materials

The following are available online. Figure S1. ^1^H-NMR (300 MHz, DMSO-*d*_6_) spectrum of cocrystal [(**1,4-bd**)·(**4,4′-bpe**)]_n_; Figure S2. ^1^H-NMR (300 MHz, DMSO-*d*_6_) spectrum of cocrystal [(**1,4-bd**)·(**4,4′-bpe**)]_n_ following 55 h of UV exposure; Figure S3. ^1^H-NMR (300 MHz, DMSO-*d*_6_) spectrum of isolated *rctt*-4,4′-tpcb from [(**1,4-bd**)·(**4,4′-bpe**)]_n_; Figure S4. ^1^H-NMR (300 MHz, DMSO-*d*_6_) spectrum of cocrystal [(**1,4-bd**)·(**3,3′-bpe**)]_n_; Figure S5. ^1^H-NMR (300 MHz, DMSO-*d*_6_) spectrum of [(**1,4-bd**)·(**3,3′-bpe**)]_n_ following 23 h of UV exposure.; Figure S6. ^1^H-NMR (300 MHz, CDCl_3_) spectrum of isolated *rctt*-**3,3′-tpcb** from [(**1,4-bd**)·(**3,3′-bpe**)]_n_.; Figure S7. Powder X-ray diffractogram of (*rctt-***3,3′-tpcb**)·(H_2_O) (top, blue) compared to the simulated pattern generated from single-crystal X-ray data (bottom, black); Figure S8. Powder X-ray diffractograms of [(**1,4-bd**)·(**4,4′-bpe**)]_n_ generated through dry grinding (top, black) compared to simulated from single-crystal X-ray diffraction data (blue). Simulated patterns of pure **1,4-bd** and **4,4-bpe** reproduced from TELXAJ and AZSTBB, respectively; Figure S9. Powder X-ray diffractograms at 50% catalyst loading of **1,4-bd** to generate [(**1,4-bd**)·(**4,4′-bpe**)]_n_ (top, blue) compared to the simulated patters of **1,4-bd** (middle, black) and [(**1,4-bd**)·(**4,4′-bpe**)]_n_ (bottom, black). Simulated pattern of pure **1,4-bd** reproduced from TELXAJ; Figure S10. ^1^H-NMR (300 MHz, DMSO-*d*_6_) spectra monitoring the photoreactivity of [(**1,4-bd**)·(**4,4′-bpe**)]_n_ at 20 mol.% catalyst loading of **1,4-bd** over 100 h of UV exposure. Total UV-exposure time (t) indicated with each-NMR; Figure S11. Powder X-Ray diffractograms of solid-state catalysis experiments with 20 mol.% loading of **1,4-bd** with **4,4′-bpe**; and Figure S12. ^1^H-NMR (300 MHz, CDCl_3_) spectrum of sublimed **1,4-bd**.
