# Supplementary material for: A Divergent Alkyne Diol Directs [2 + 2] Photoreactivity in the Solid State: Cocrystal, Supramolecular Catalysis, and Sublimation Effects
Source: Molecules. 2019 Aug 22;24(17):3059. doi: 10.3390/molecules24173059 (PMC6749457; doi:10.3390/molecules24173059)
Supplement: Supplementary file 1 [file molecules-24-03059-s001.pdf]

Supplementary Materials

A Divergent Alkyne Diol Directs [2+2] Photoreactivity in the Solid State: Cocrystal, Supramolecular Catalysis, and Sublimation Effects

Shalisa M. Oburn, Jay Quentin, and Leonard R. MacGillivray[1,*]

[1] Department of Chemistry, University of Iowa, Iowa City, IA, 52242, USA.
* Correspondence: len-macgillivray@uiowa.edu

Contents:

Figure S1. ^{1}H NMR (300 MHz, DMSO-d_6) spectrum of cocrystal [(**1,4-bd**)·(**4,4′-bpe**)]$_n$.

Figure S2. ^{1}H NMR (300 MHz, DMSO-d_6) spectrum of cocrystal [(**1,4-bd**)·(**4,4′-bpe**)]$_n$ following 55 h of UV-exposure.

Figure S3. ^{1}H NMR (300 MHz, DMSO-d_6) spectrum of isolated *rctt*-**4,4′-tpcb** from [(**1,4-bd**)·(**4,4′-bpe**)]$_n$.

Figure S4. ^{1}H NMR (300 MHz, DMSO-d_6) spectrum of cocrystal [(**1,4-bd**)·(**3,3′-bpe**)]$_n$.

Figure S5. ^{1}H NMR (300 MHz, DMSO-d_6) spectrum of [(**1,4-bd**)·(**3,3′-bpe**)]$_n$ following 23 h of UV-exposure.

Figure S6. ^{1}H NMR (300 MHz, $CDCl_3$) spectrum of isolated *rctt*-**3,3′-tpcb** from [(**1,4-bd**)·(**3,3′-bpe**)]$_n$.

Figure S7. Powder X-ray diffractogram of (*rctt*-**3,3′-tpcb**)·(H_2O) (top, blue) compared to the simulated pattern generated from single-crystal X-ray data (bottom, black).

Figure S8. Powder X-ray diffractograms of [(**1,4-bd**)·(**4,4′-bpe**)]$_n$ generated through dry grinding (top, black) compared to simulated from single-crystal X-ray diffraction data (blue). Simulated patterns of pure **1,4-bd** and **4,4-bpe** reproduced from TELXAJ[1] and AZSTBB[2], respectively.

Figure S9. Powder X-ray diffractograms at 50% catalyst loading of **1,4-bd** to generate [(**1,4-bd**)·(**4,4′-bpe**)]$_n$ (top, blue) compared to the simulated patters of **1,4-bd** (middle, black) and [(**1,4-bd**)·(**4,4′-bpe**)]$_n$ (bottom, black). Simulated pattern of pure **1,4-bd** reproduced from TELXAJ[1]

Figure S10. ^{1}H NMR (300 MHz, DMSO-d_6) spectra monitoring the photoreactivity of [(**1,4-bd**)·(**4,4′-bpe**)]$_n$ at 20 mol. % catalyst loading of **1,4-bd** over 100 h of UV-exposure. Total UV-exposure time (t) indicated with each NMR.

Figure S11. Powder X-Ray diffractograms of solid-state catalysis experiments with 20 mol. % loading of **1,4-bd** with **4,4′-bpe**.

Figure S12. ^{1}H NMR (300 MHz, $CDCl_3$) spectrum of sublimed **1,4-bd**.

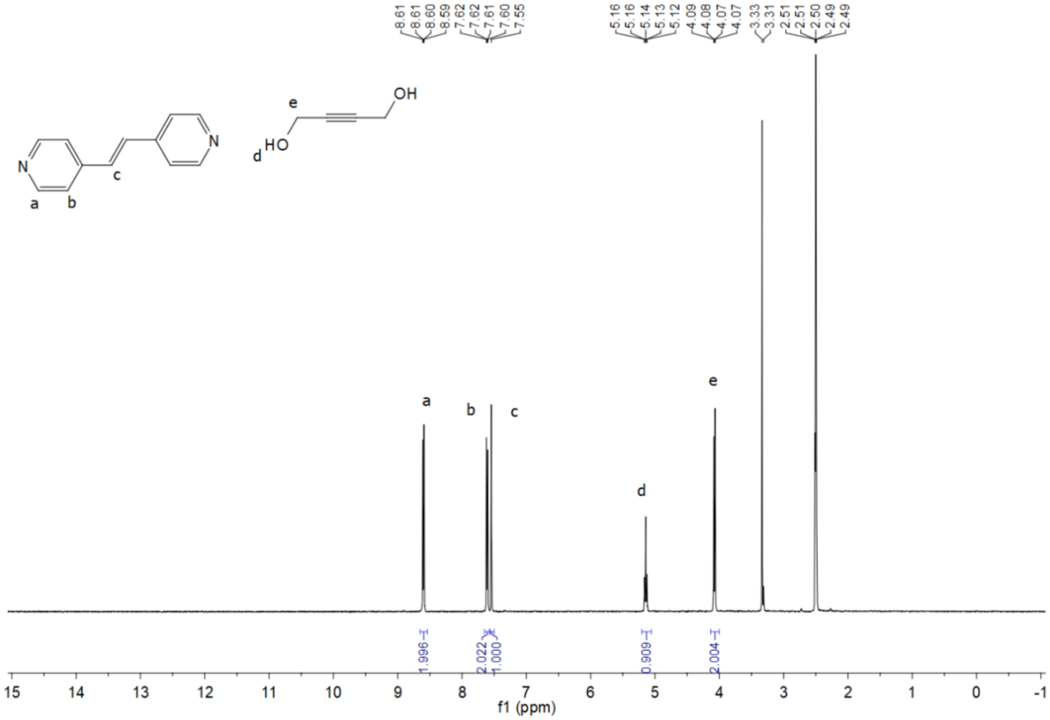

Figure S1. ^{1}H NMR (300 MHz, DMSO-*d*6) spectrum of cocrystal [(**1,4-bd**)·(**4,4′-bpe**)]n.

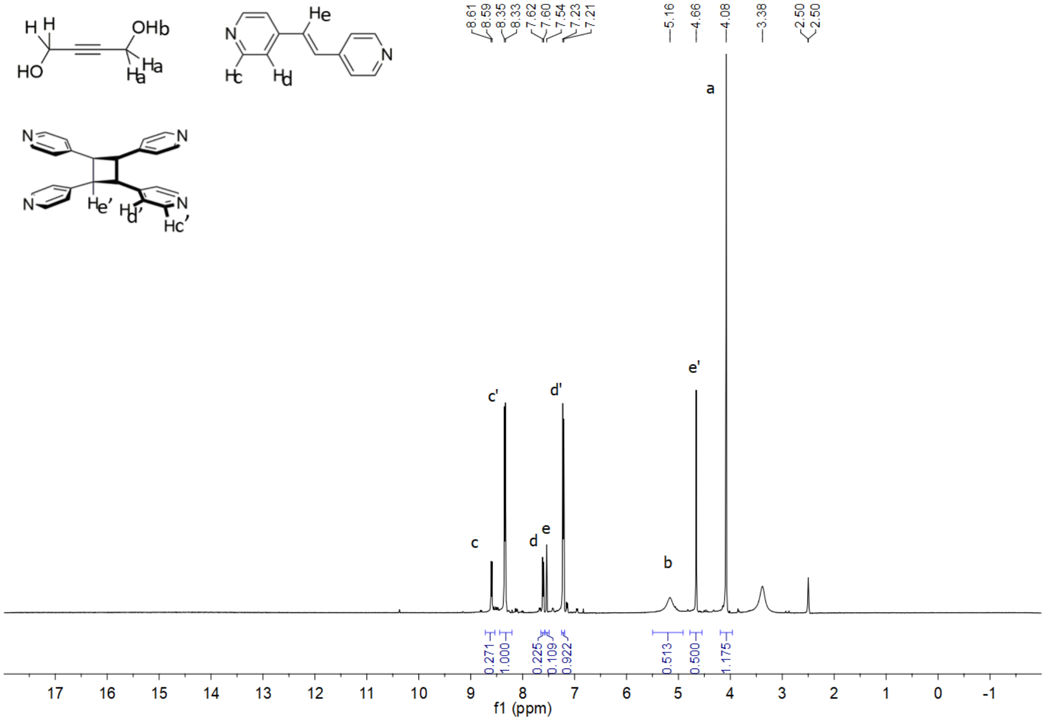

Figure S2. ^{1}H NMR (300 MHz, DMSO-*d*6) spectrum of cocrystal [(**1,4-bd**)·(**4,4′-bpe**)]n following 55 h of UV-exposure.

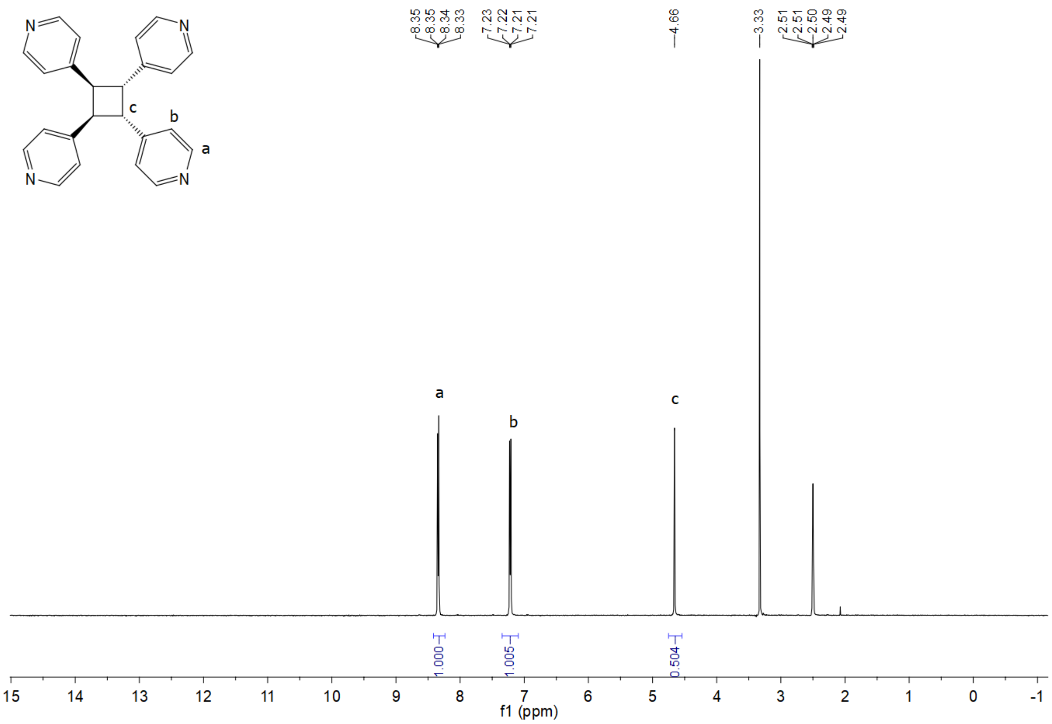

Figure S3. ^{1}H NMR (300 MHz, DMSO-d_6) spectrum of isolated *rctt*-**4,4′-tpcb** from [(**1,4-bd**)·(**4,4′-bpe**)]n.

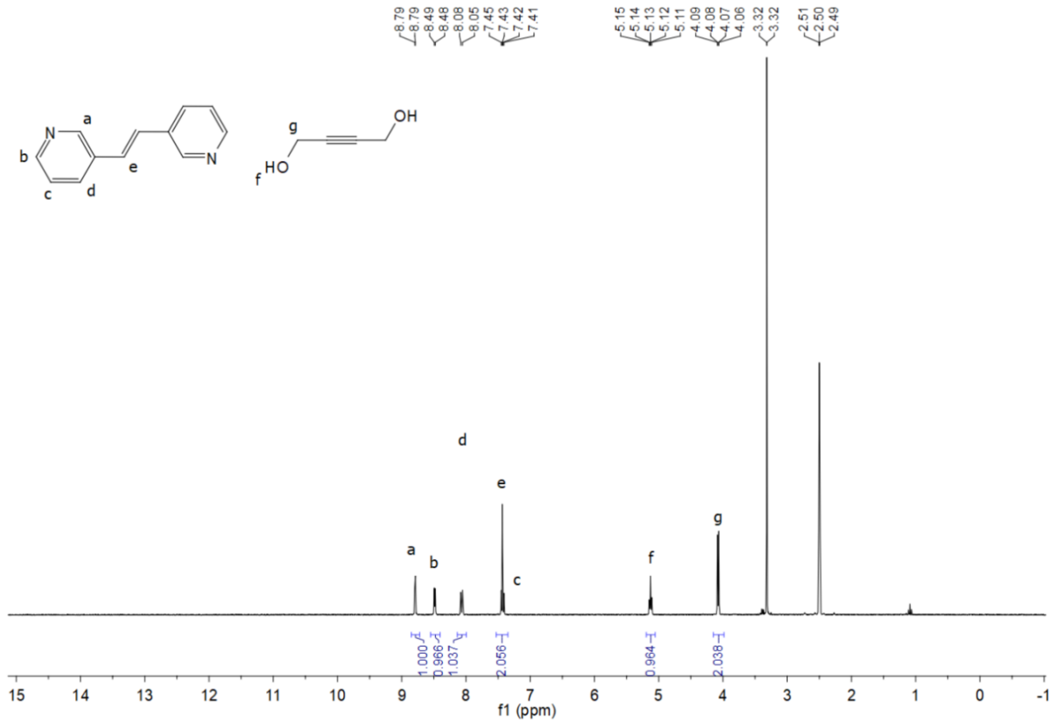

Figure S4. ^{1}H NMR (300 MHz, DMSO-d_6) spectrum of cocrystal [(**1,4-bd**)·(**3,3′-bpe**)]n.

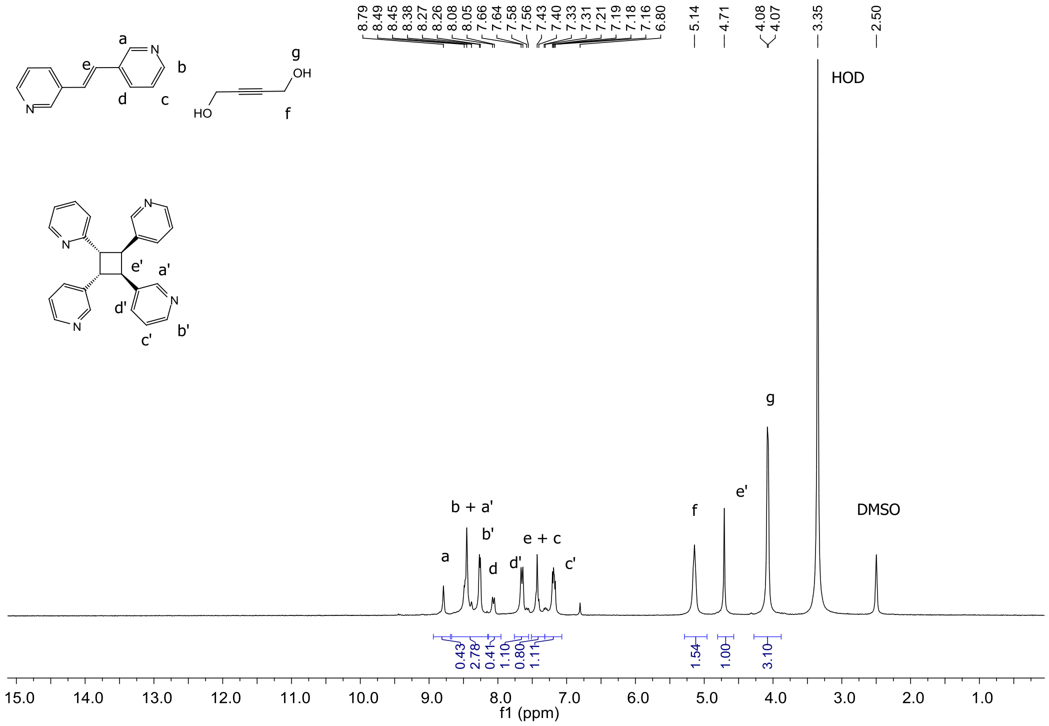

Figure S5. ^{1}H NMR (300 MHz, DMSO-d_6) spectrum of [(**1,4-bd**)·(**3,3′-bpe**)]$_n$ following 23 h of UV-exposure.

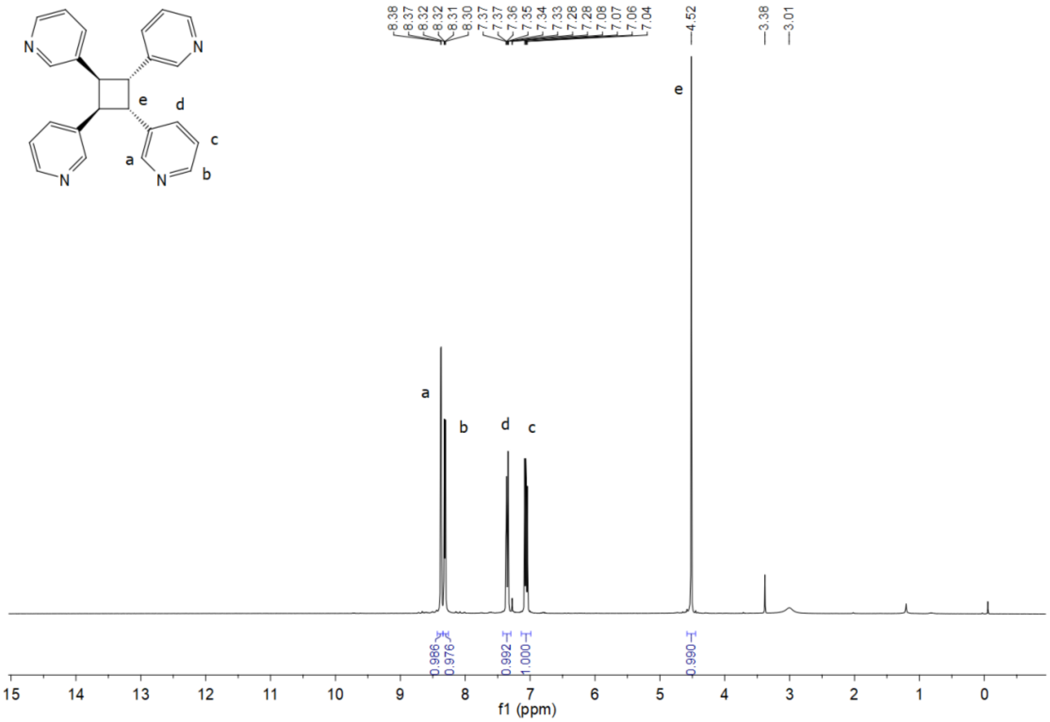

Figure S6. ^{1}H NMR (300 MHz, CDCl$_3$) spectrum of isolated *rctt*-**3,3′-tpcb** from [(**1,4-bd**)·(**3,3′-bpe**)]$_n$.

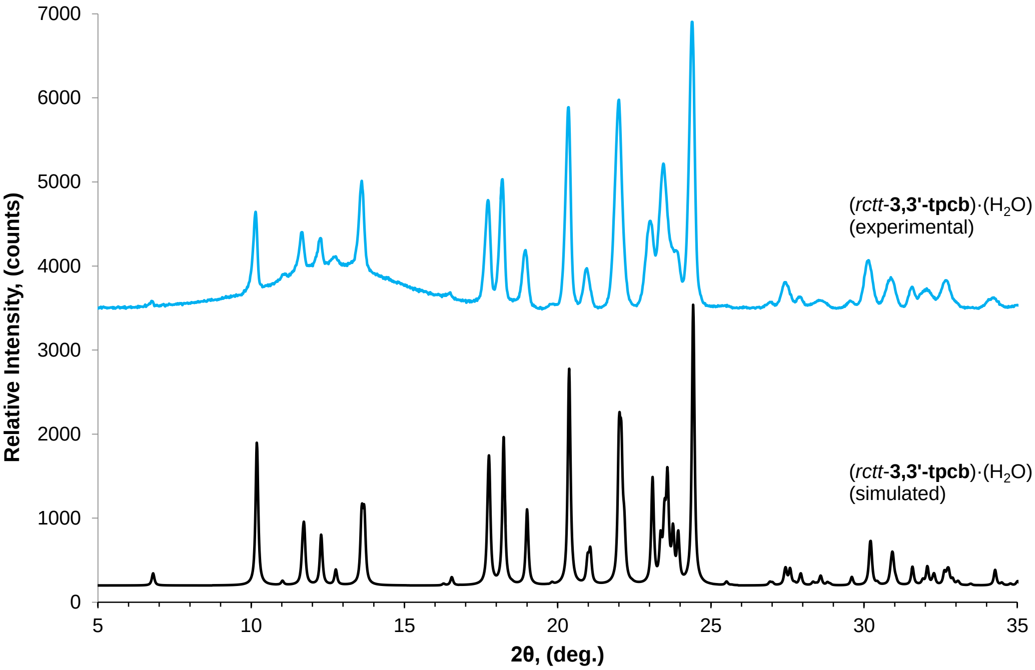

Figure S7. Powder X-ray diffractogram of (*rctt*-**3,3′-tpcb**)·(H_2O) (top, blue) compared to the simulated pattern generated from single-crystal X-ray data (bottom, black).

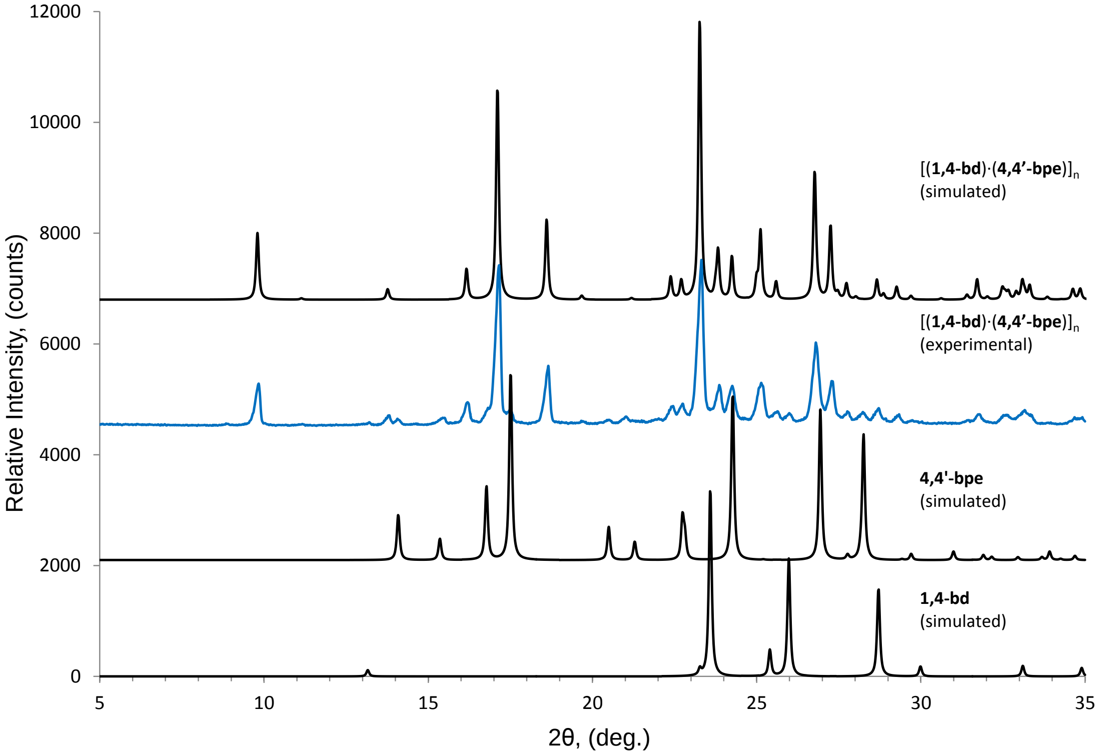

Figure S8. Powder X-ray diffractograms of [(**1,4-bd**)·(**4,4′-bpe**)]$_n$ generated through dry grinding (top, black) compared to simulated from single-crystal X-ray diffraction data (blue). Simulated patterns of pure **1,4-bd** and **4,4-bpe** reproduced from TELXAJ[1] and AZSTBB[2], respectively.

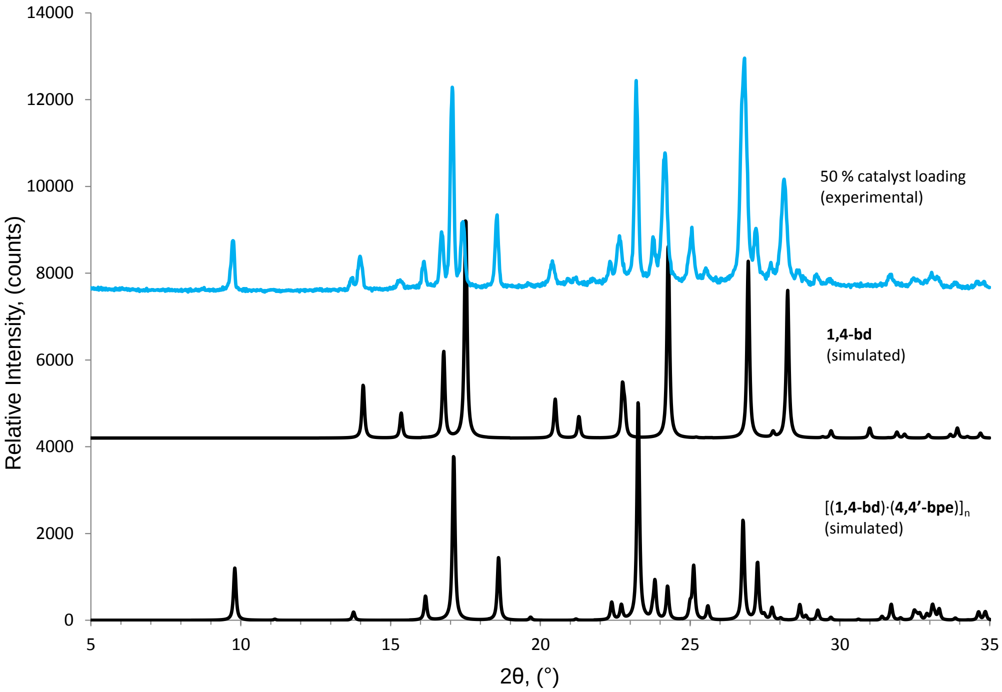

Figure S9. Powder X-ray diffractograms at 50% catalyst loading of **1,4-bd** to generate [(**1,4-bd**)·(**4,4′-bpe**)]$_n$ (top, blue) compared to the simulated patters of **1,4-bd** (middle, black) and [(**1,4-bd**)·(**4,4′-bpe**)]$_n$ (bottom, black). Simulated pattern of pure **1,4-bd** reproduced from TELXAJ[1]

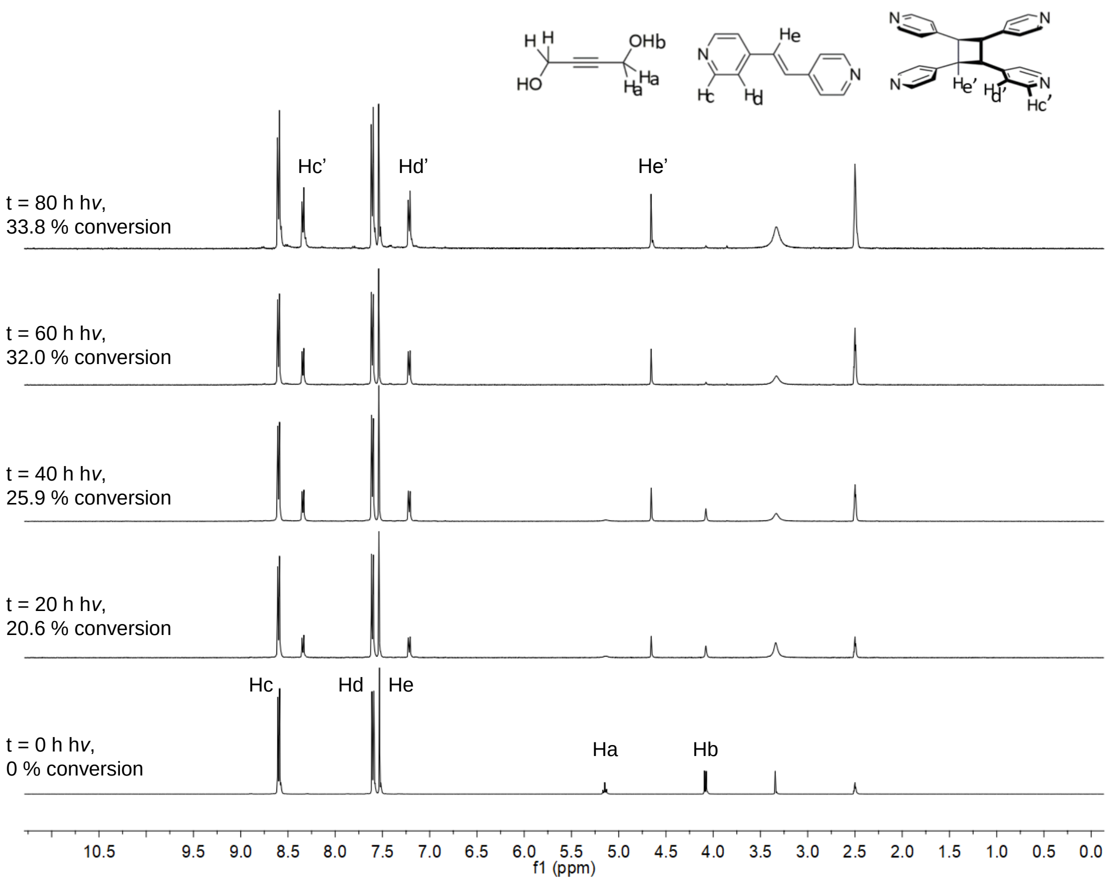

Figure S10. ^{1}H NMR (300 MHz, DMSO-d_6) spectra monitoring the photoreactivity of [(**1,4-bd**)·(**4,4′-bpe**)]$_n$ at 20 mol. % catalyst loading of **1,4-bd** over 100 h of UV-exposure. Total UV-exposure time (t) indicated with each NMR.

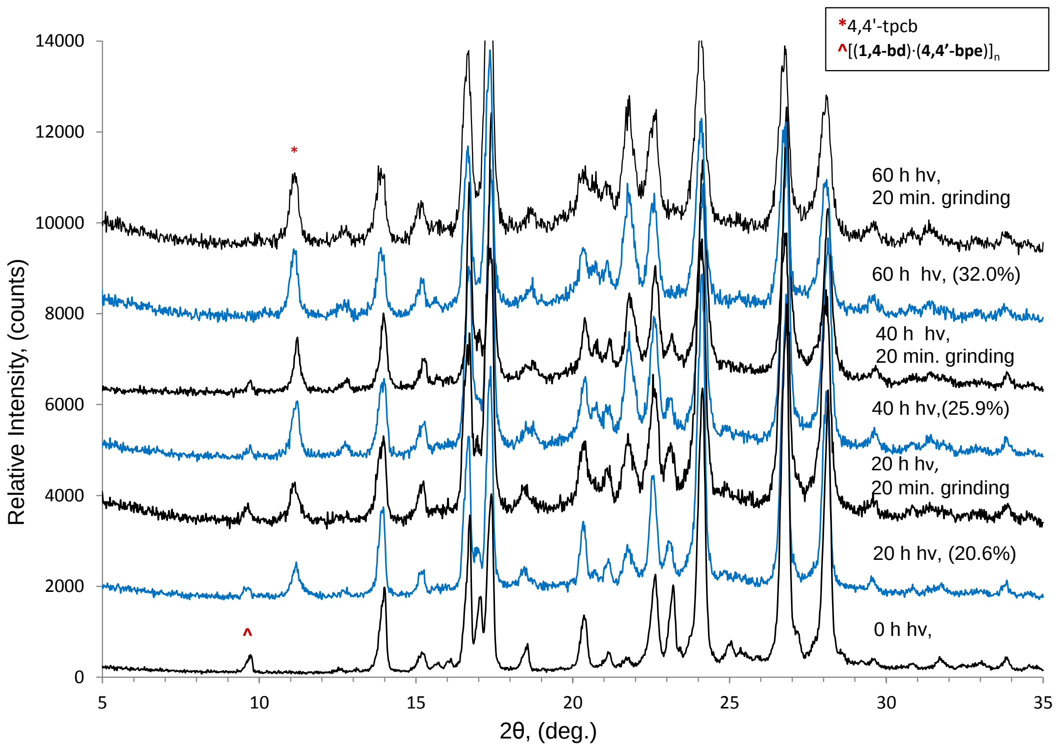

Figure S11. Powder X-Ray diffractograms of solid-state catalysis experiments with 20 mol. % loading of **1,4-bd** with **4,4′-bpe**.

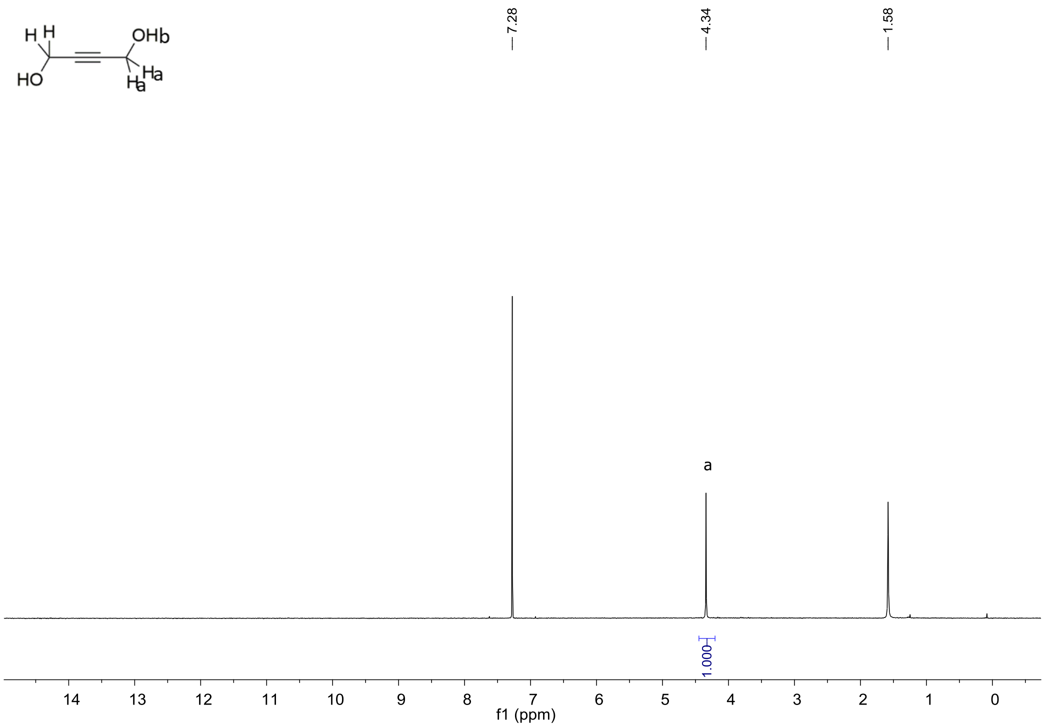

Figure S12. ^{1}H NMR (300 MHz, $CDCl_3$) spectrum of sublimed **1,4-bd**.

References

1. Steiner, T., 2-Butyne-1,4-diol. *Acta Crystallogr. Sec. C* **1996,** 52, (11), 2885-2887.
2. Vansant, J.; Smets, G.; Declercq, J. P.; Germain, G.; Van Meerssche, M., Azastilbenes. 1. Synthesis, characterization, and structure. *J. Org. Chem.* **1980,** 45, (9), 1557-1565.
